# Evaluation of NADPH Oxidase (NOX) Activity by nitro Blue Tetrazolium (NBT) Test in SLE Patients

**DOI:** 10.31138/mjr.34.2.163

**Published:** 2023-06-30

**Authors:** Ritasman Baisya, Shiva Krishna Katkam, Sreejitha KS, Phani Kumar Devarasetti, Vijay Kumar Kutala, Liza Rajasekhar

**Affiliations:** 1 Clinical Immunology and Rheumatology, Nizams Institute of Medical Sciences (NIMS), Hyderabad, India,; 2 Clinical Pharmacology & Therapeutics, Nizam’s Institute of Medical Sciences, Hyderabad Telangana, India

**Keywords:** SLE, nitro-blue tetrazolium (NBT), Reactive oxygen species (ROS), BILAG score, SLEDAI score

## INTRODUCTION

Innate immunity plays a crucial part in initiating and perpetuating the disease process in SLE. Recent studies have pushed the neutrophil, an essential component of the innate immunity, to the forefront of the pathogenesis of SLE.^1^ Nicotinamide adenine dinucleotide phosphate (NADPH) oxidase (NOX2), constitutively expressed in neutrophils, plays a pivotal role in producing cytosolic reactive oxygen species (ROS).^2^ ROS-mediated stress has been implicated in various autoimmune and immunodeficiency diseases, including SLE.

There is conflicting evidence in the literature on basal and stimulated cytoplasmic.

ROS production in SLE patients. Some studies have reported an increased ROS production^3^ in SLE patients, while others showed a negative association between SLE and ROS.^4^ Production of ROS depends on neutrophilic NOX2 activity. It is unclear what happens to NOX2 level in SLE and whether it varies in different disease activity states. Recent studies on exacerbation of lupus in NOX2 or its subunit deficient lupusprone mice^5,6^ brought forth the idea that SLE patients, particularly those with high disease activity, might have less NOX2 level than usual. Since flares characterize the lupus disease course, the NOX2 levels may be low due to increased disease activity.

Measuring the quantity of cytosolic ROS with nitro blue tetrazolium (NBT) test, is an indirect measure of NOX2, as the latter is needed for their production and would therefore give an idea of any correlation between ROS and disease activity in SLE.

## OBJECTIVE

The primary objective of the study was to compare cytosolic reactive oxygen species (ROS) production (both basal and after stimulation with PMA) using Nitro blue tetrazolium (NBT) assay in high versus low disease activity states in SLE patients and healthy controls. Other objectives were to compare the level of ROS production with disease activity, clinical and serological status and different outcome measures - SLE disease activity index-2K (SLEDAI-2K), Physician global assessment by visual analogue scale (PhGA -VAS), British Isles Lupus assessment group index (BILAG scores).

## METHODS

### Study Population

This was a cross-sectional, hospital-based study including consecutive SLE patients visiting Rheumatology outdoor or indoor in Nizam’s Institute of Medical Sciences, Hyderabad fulfilling SLE International Collaboration Classification (SLICC criteria - 2012)^7^ or American College of Rheumatology criteria for SLE (1997)^8^ conducted from November 2019 to March 2020. The patients were divided into two groups based on disease activity (high and low) with a SLEDAI-2K cut-off of six (≥ 6 - high, <6 -low). Age & sex-matched healthy subjects were included as controls. Acutely ill patients (with sepsis, malignancy, cardiovascular decompensation), those with fever, any evidence of infection in the previous month and neutropenia during data collection were excluded from the study. Informed consent was taken for all subjects. Ethical approval was taken as per declaration of Helsinki (EC/NIMS/2413/2019, 41^st^ ESGS No: 882/2019)

### Clinical History and Investigation

Demographic history, clinical features, serology (dsDNA, complement), history of past infection, treatment history were recorded for all cases. Disease activity was measured by SLEDAI-2K, BILAG index and Physician Global Assessment (PhGA) measured on a 3 cm visual analog scale (VAS).

### Isolation of Neutrophils from peripheral blood

A volume of 6 ml of peripheral blood was collected from subjects in an EDTA vial and immediately sent for processing. The sample was diluted with 1x PBS (pH 7.4) buffer without calcium & magnesium ions at a 1:1 ratio. Diluted blood was layered over 4–5 ml of Ficoll-paque solution and centrifuged at 2000 rpm for 20 min at room temperature. After centrifugation, distinct layers were observed, with plasma, peripheral blood mononuclear cells (PBMC), Lyphodex solution, and pellets of sedimented red blood cells (RBC) with granulocytes. The first three layers of plasma, PBMC and Lyphodex solution were discarded to obtain RBC pellets with granulocytes. The RBC pellets were immediately suspended in 2 ml of 1X PBS buffer for further processing. Dextran sedimentation was performed with a 6% dextran solution to separate the neutrophils from RBC sediment. Neutrophil-rich supernatant was collected and centrifuged for 10 minutes. Then RBC lysis was done to remove residual RBCs as supernatant and a white pellet consisting of granulocytes was obtained.

### Morphology verification

**Supplementary Figure 1s.**
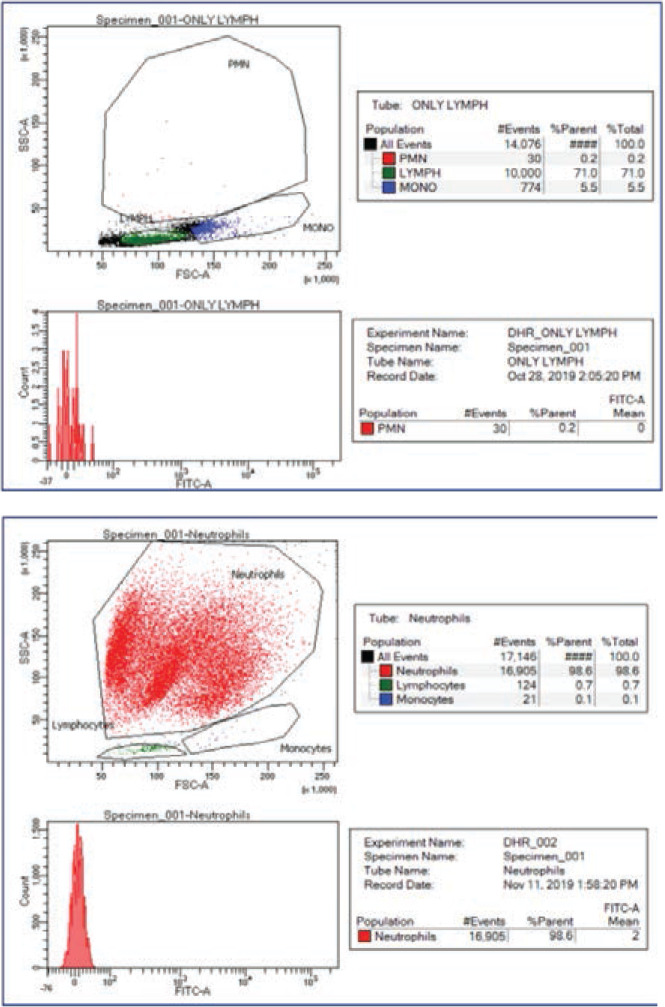
Analysis of neutrophil Purity by flow cytometry. A forward-scatter versus side scatter dot plot was used to evaluate neutrophil purity.

After lysis, morphological examination and trypan blue exclusion test were performed to determine cell count and purity of neutrophils. Freshly isolated neutrophils were stained with 0.4% trypan blue and mixed under countess™ automated cell counter. The morphological observation revealed that >95% of cells isolated were live neutrophils. Analysis of neutrophil purity was performed by flow cytometry. (**Supplementary Figure 1s**)

### NBT reduction

**Supplementary Figure 2s.**
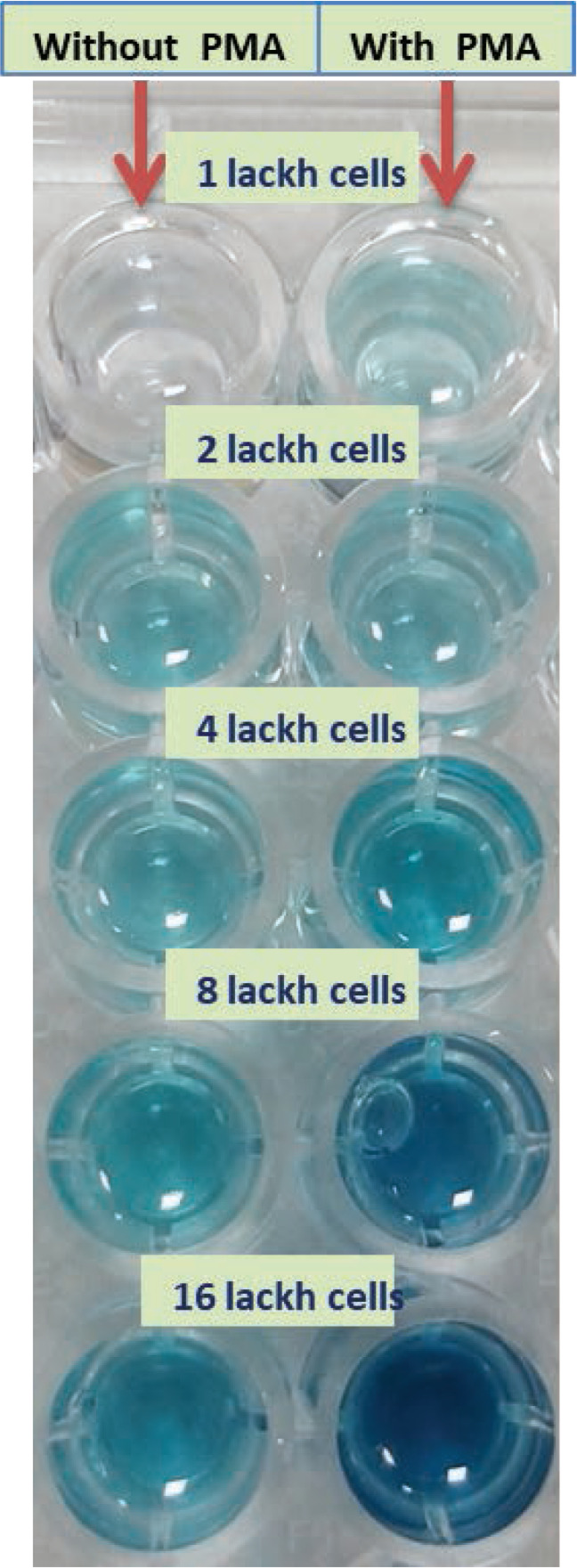
NBT reduction with different cell numbers (1, 2, 4, 8, and 16 lackh cells/well without and with PMA.

**Supplementary Figure 3s.**
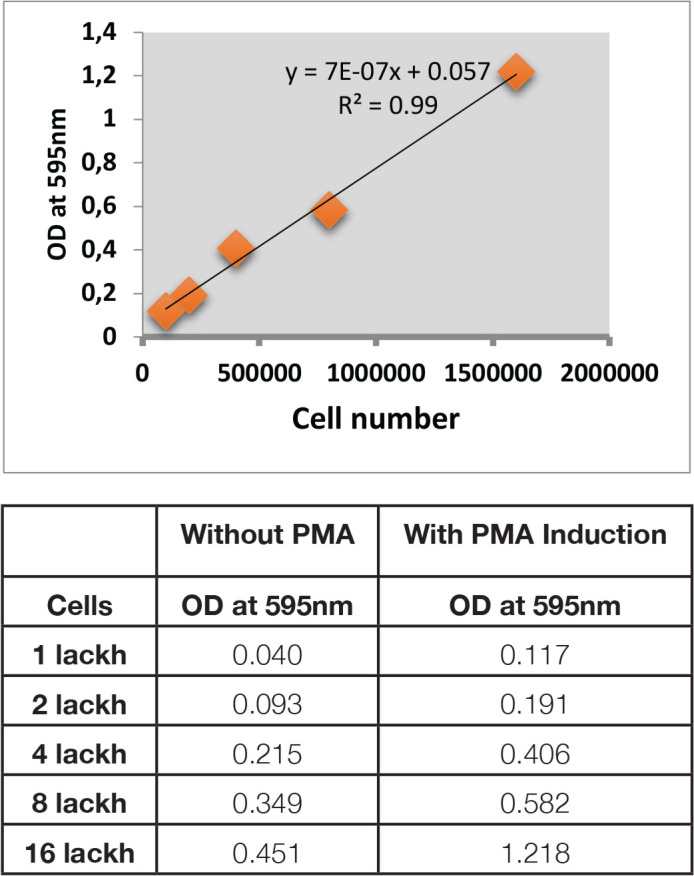
OD value (595 nm) with different neutrophil count (with and without PMA).

The Nitro blue tetrazolium (NBT) 0.1% solution was prepared by adding 1mg of NBT powder (Sigma) to 10mL of PBS (pH 7.2) and filtered with a 0.2mm filter. NBT assay was performed using freshly isolated neutrophils (1x10 6 cells) suspended in PBS. Phorbol myristate acetate (PMA) was used for stimulation of the neutrophils. The cells were incubated with 100μl of NBT (0.1%) and 1μl of PMA (2.5mg/ml) for 1 hour. A replicate set of cells without PMA were also incubated for an hour. The NBT gets imbibed into the cell cytoplasm and reacts with super-oxide radicals forming water-insoluble formazan crystals, indicating the neutrophils’ ability to reduce NBT; resulting in a purple-blue colour. The insoluble blue formazan was solubilized leaving a solution with turquoise-blue colour. This turquoise-blue colour intensity in the cells was assessed using a microplate reader at 595 nm to quantify the final product. (**Supplementary Figures 2s, 3s**)

### Statistical analysis

Mean value of basal, stimulated, and change in ROS production as measured by NBT were compared between SLE cases and healthy control with student unpaired t-test. Kendall-Tau correlation matrix was performed to detect the influence of disease activity on basal, stimulated, and change in ROS production. Pooled analysis of basal, stimulated, and change in ROS production was done for different groups, including case and healthy controls. Basal and stimulated ROS levels were compared in each category (A, B, C, D, E) of BILAG renal, mucocutaneous, and hematologic domains by ANOVA test. IBM SPSS 2021 version was used for statistical analysis.

## RESULTS

### Demographic characteristics

The total number of SLE patients and controls included were 73 and 46, with a mean age of 28.5 & 35 years, respectively. Mean SLEDAI was 13.47 ± 8.89, with low disease activity (SLEDAI<6) seen in 25 (34.25%) and high disease activity in 47 (64.38%) patients. Mean physician global assessment by VAS scale was 1.498. At the time of sample collection, BILAG -A in the mucocutaneous domain was seen in 12 (16.43%), neurology domain in 9 (12.33%), musculoskeletal domain in 3 (4.11%), cardiorespiratory domain in 7 (9.58%), gastrointestinal domain in 3 (4.11%), ophthalmological domain in 1, renal domain in 28 (38.36%), and haematological domain in 5 (6.85%) patients. Damage from SLE in the form of avascular necrosis (AVN) hip was seen in 3 (4.11%) patients and pulmonary arterial hypertension (PAH) in 4 (5.58%) patients (**Table 1**).

**Table 1.** Demographic and clinical characteristics of the studied SLE patients and controls.

**Demographic variable**	**Cases (n=73) (%)**	**Controls (n=46) (%)**
Female	66 (90.4)	31 (67.3)
Mean age	28.4 years	35.0 years
Comorbidity	22 (30.14)	0
Low & high disease activity	25/73, 48/43	
Low dose steroid	29(39.7)	
PGA (VAS <1)	26 (35.6)	
Ever leucopenia	37 (50.7)	
dsDNA ever positive	48 (65.8)	
Complement ever low	58 (79.5)	
Past tuberculosis	9 (12.3)	
BILAG A mucocutaneous	12 (16.4)	
BILAG A neurological	9 (12.3)	
BILAG A musculoskeletal	3 (4.1)	
BILAG A cardiorespiratory	7 (9.6)	
BILAG A gastrointestinal	3 (4.1)	
BILAG A renal	28 (38.4)	
BILAG A haematological	5 (6.9)	
SLE damage - AVN hip	3 (4.1)	
SLE damage – PAH	4 (5.6)	

### NBT reduction in case vs control

**Figure 1.**
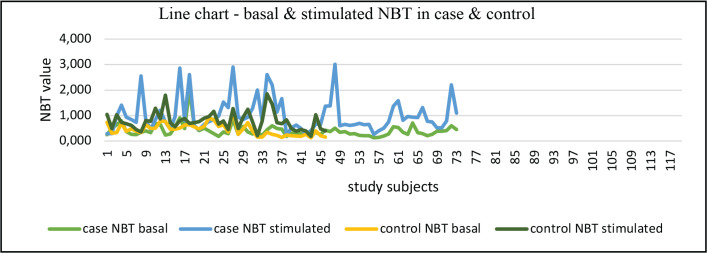
Line chart – basal & stimulated NBT in case and control.

SLE subjects showed significant NBT reduction (1.03±0.67) on stimulation compared to controls (0.79±0.36) (p=0.03). However, basal NBT reduction did not differ between cases (0.42±0.25) and control (0.45±0.24) (p=0.52) (**Table 2, Figure 1**).

**Table 2.** Basal and stimulated NBT reduction (mean ± SD) in case versus control.

**Study group**	**SLE cases**	**Healthy controls**	**p-value**
NBT (Basal)	0.42 ± 0.25	0.45 ± 0.24	0.52
NBT (Stimulated)	**1.03 ± 0.67**	**0.79 ± 0.36**	**0.03**

SLE patients showed higher stimulated NBT reduction compared to control (p – 0.03), basal NBT is lower in cases but not statistically significant.

### NBT reduction according to disease activity

**Figure 2.**
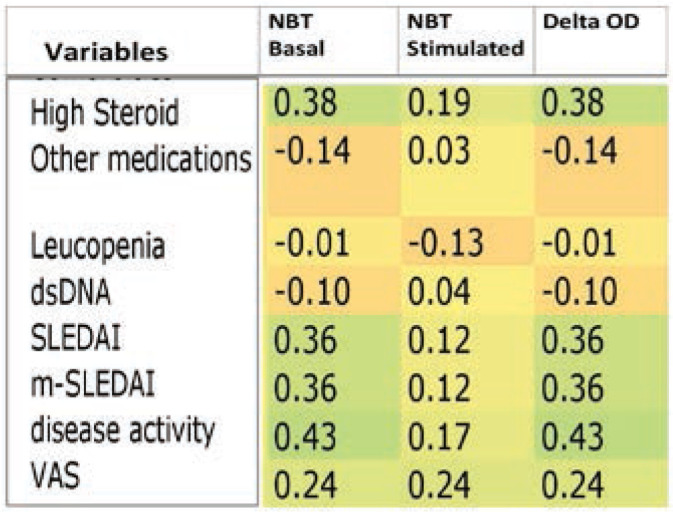
Kendall–Tau matrix - SLEDAI, m-SLEDAI (Mexican -SLEDAI/clinical SLEDAI) are strongly associated with higher basal and ΔNBT (Del OD) values but not with stimulated NBT values. High dose steroid: >−10 mg prednisolone or equivalent/day. Other medications include any steroid sparing immunosuppressive – azathioprine, cyclophosphamide, mycophenolate, etc. The matrix represents correlation coefficients of pairwise variables. The red background depicts inverse association, while the green background depicts positive association.

The basal NBT reduction was significantly higher in cases with high disease activity than those with low disease activity (0.46 ± 0.29 vs 0.31 ± 0.11, p=0.015). Stimulated NBT reduction in patients with high disease activity compared to low disease activity was not significantly different. Kendall-Tau correlation matrix showed the SLEDAI-2K was strongly associated with higher basal and ΔROS (increment in ROS) production (**Figure 2**). However, levels of dsDNA or complements did not show any significant association.

**Figure 3.**
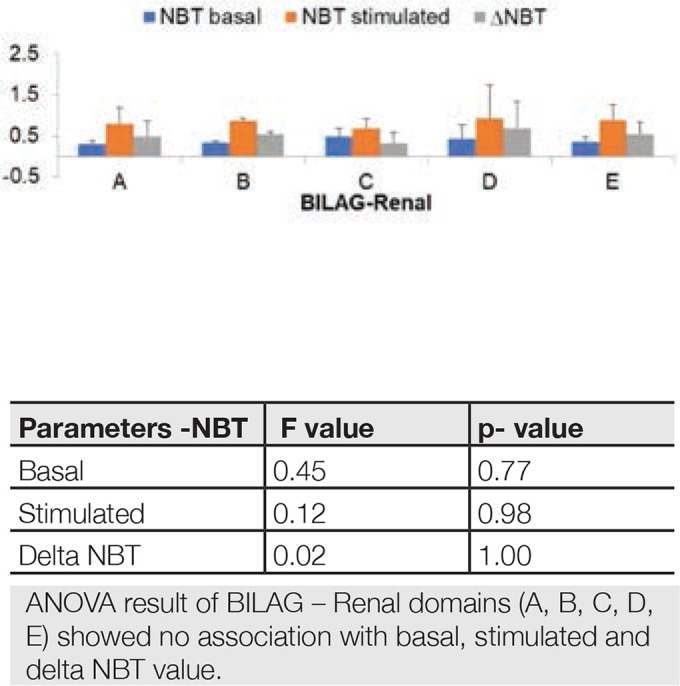
Comparison of basal, stimulated NBT reduction for each category (A, B, C, D, E) of renal BILAG domain.

Patients with lupus nephritis showed higher basal NBT reduction than without nephritis (0.43±0.26 vs 0.30±0.09, p=0.0001) (**Table 3**), but there was no statistically significant difference between individual renal BILAG domains (**Figure 3**). PhGA-VAS scale also showed a significant correlation with both basal and stimulated ROS production (p=0.04 & 0.003, respectively).

**Table 3.** Basal and stimulated NBT reduction (mean ± SD) in lupus nephritis patients.

**Study Group**	**NBT Basal**	**NBT stimulated**
**Lupus nephritis present**	0.43 ± 0.26	1.05 ± 0.69
**Lupus nephritis absent**	0.30 ± 0.09	0.92 ±0.56
**p-value**	**<0.0001**	0.21

Patients with lupus nephritis showed higher basal and stimulated NBT compared to those without nephritis. basal NBT is statistically significant.

**Figure 4.**
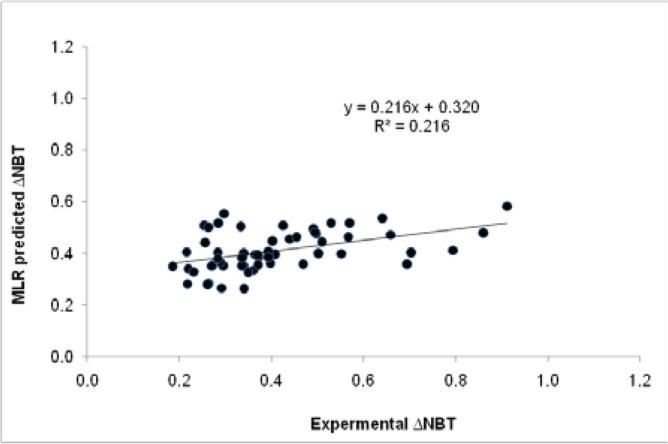
Age, gender, co-morbidities, high steroid dose, other medications, leukopenia, dsDNA, past infections explain 21.6% variability in ΔNBT. The association of ROS with using demographic and clinical variables, multiple linear regression analysis was performed. Age, gender, comorbidities, high steroid dose, other medications, leukopenia, dsDNA, past infections explained 21.6% variability in ΔNBT (increment in ROS levels).

The association of ROS with demographic and clinical variables, using multiple linear regression analysis was performed. Age, gender, comorbidities, high steroid dose, other medications, leukopenia, dsDNA and past infections explained 21.6% variability in ΔNBT (increment in ROS levels). (**Table 4, Figure 4**)

**Table 4.** Multiple Linear Regression - Ordinary Least Squares showing association of ΔNBT values with demographic and clinical variables.

**Variable**	**Parameter**	**S.D.**	**T-STAT H0: parameter = 0**	**2-tail p-value**	**1-tail p-value**
**Age**	+0.001	0.002	+5.3460e-01	0.59	0.29
**Gender**	−0.075	0.074	−1.0080e+00	0.31	0.15
**Co-morbidities**	−0.086	0.050	−1.7280e+00	0.09	0.04
**High steroid dose**	**+0.116**	**0.046**	**+2.5010e+00**	**0.01**	**0.007**
**Other medications**	+0.023	0.049	+4.7560e-01	0.63	0.31
**Leukopenia**	−0.020	0.046	−4.4560e-01	0.65	0.32
**ds DNA**	−0.039	0.051	−7.5590e-01	0.45	0.22
**Past infections**	+0.032	0.045	+7.2460e-01	0.47	0.23

**Supplementary Figure 4s.**
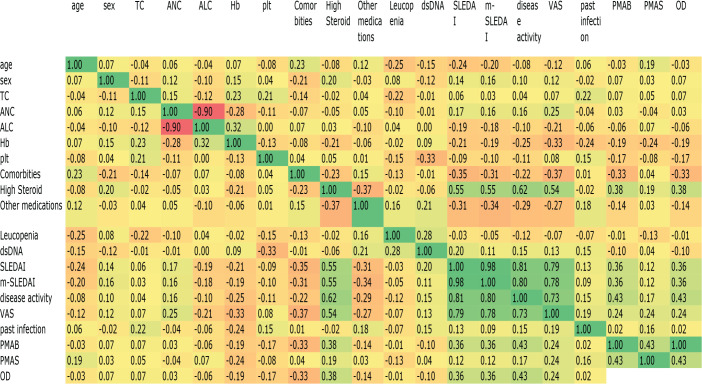
Kendall-Tau correlation matrix. The matrix represents correlation coefficients of pair-wise variables. The red background depicts inverse association while the green background depicts positive association. NBT basal levels and ΔNBT values are not influenced by age, gender, total leukocyte count, neutrophil count, lymphocyte count, hemoglobin, and platelet count. However, high steroid dose increases basal NBT and ΔNBT values while comorbidities decrease the basal and ΔNBT. The SLEDAI, m-SLEDAI and disease activity are strongly associated with higher basal and ΔNBT values. Stimulated NBT values are not affected by demographic, haematological, and clinical variables investigated.

Kendall-Tau correlation matrix also showed the basal and ΔROS (increment in ROS) levels were not influenced by age, gender, total leukocyte count, neutrophil count, lymphocyte count, haemoglobin, and platelet count. However, high steroid dose increased basal ROS and ΔROS values while comorbidities decreased the basal and ΔROS. (**Supplementary Figure 4s**)

## DISCUSSION

In the present study, using the NBT test, we showed that SLE subjects had higher stimulated cytosolic ROS production than healthy controls. Basal ROS generation significantly increased in high disease activity state compared to low disease activity. In patients with lupus nephritis, there was a significantly higher basal ROS level. Age, gender, comorbidities, high steroid dose, other medications, leukopenia, dsDNA, and past infections explain one-fifth of the increase in ROS with stimulation. Perazzio et al.^3^ have reported increased oxidative burst in SLE patients both at basal and stimulated state compared to healthy controls. Our study confirms their observation. SLE neutrophils may be primed for respiratory burst due to the existing inflammatory milieu. Though several groups have previously shown increased ROS production by SLE neutrophils after stimulation,^9–13^ there is contradictory evidence in the literature regarding the oxidative status of SLE neutrophils upon stimulation.

Bengtsson et al.^4^ reported low respiratory burst using Phago-Burst assay and 2,7-dichlorofluorescein-diace-tate (DCFH-DA) test on a cohort of Swedish SLE patients compared to controls. However, this cohort of patients had lower disease activity, fewer patients with nephritis, and a greater proportion of patients with organ damage. These factors could explain the low respiratory burst observed. Marzocchi et al.^14^ have also reported a significant decrease in the oxidative burst of neutrophils in Brazilian patients with active SLE than controls. Here they examined the complement and FcϒR mediated oxidative burst rather than PMA stimulated oxidative burst. It was found that FcϒR levels were depleted in the inflammatory milieu, and this assessed a pathway of ROS generation different from our study.

In another study by Philip et al.,^15^ using the NBT test, no difference in the oxidative burst was demonstrated between SLE cases and healthy controls. A few studies have reported a negative association between SLE and ROS generation. The present study is in conformation with most of the previous studies proving a connection between SLE and ROS production.

Perazzio et al. did not find any correlation between disease activity and oxidative burst in SLE neutrophils. This finding led them to suggest that changes in ROS levels are independent of the inflammatory process. Instead, it is influenced by the ongoing subclinical immunological disorder present even in patients with apparently quiescent disease. Our results differed from the above conclusions. We have demonstrated that basal cytosolic ROS production was higher with increased disease activity with a strong positive correlation between SLEDAI and increment in ROS production on stimulation. Though in several studies, it was found that high disease activity was associated with an increased oxidative burst,^16,17^ the exact association between SLE activity and neutrophilic ROS generation is still unclear. Widespread tissue inflammatory processes could probably cause a more significant amount of ROS production even at the basal state by the neutrophils in patients with active SLE. There is no data available in literature where neutrophil oxidase activity was correlated with the BILAG score. In this study, no difference was found in basal and stimulated ROS production with BILAG scoring of mucocutaneous, renal, and haematological domains.

Our study demonstrates that lupus nephritis patients had a significantly higher ROS production in the basal state than those without nephritis. Production of ROS on stimulation is also greater in this group, even though it was not statistically significant. This finding was similar to Perazzio et al., where patients with lupus nephritis presented higher ROS production increments after stimulus with S. aureus than those without nephritis. But Phillip et al. did not find any association between nephritis and basal ROS production.

The basal oxidative status of neutrophils can be further explored using neutrophil NADPH oxidase. Campbell’s study^5^ showed that NADPH oxidase deficient mice were more prone to lupus with severe organ involvement. Jacob CO et al.^6^ reported that even haploinsufficiency of NCF-2 (neutrophil cytosolic factor 2 encoding p67^PHOX^) accelerated the development of full-blown lupus, suggesting a lupus-prone background. NBT being the measure of NADPH oxidase activity in neutrophils, it was expected that NBT basal value should be low in SLE patients compared to controls. In the Perazzio study, the basal ROS production was high in cases compared to control. Even though the mean basal NBT was low in the SLE group, it was not statistically significant in our study. This observation creates a lot of confusion about the source of ROS in SLE, whether it is only dependent on neutrophilic NOX or whether there are other factors that contribute to it. Most of the genetic studies on NOX and its component deficiency were performed in mice and proved its association with lupus exacerbation. Campbell had claimed that NOX-derived NET generation is not the primary driver in SLE, proposing instead that NOX can regulate the disease activity independent of NETosis, by increasing T reg cell and effective phagocytosis, and NOX2 deficient mice can undergo increase inflammatory cell death, like pyroptosis and necroptosis.

Limitations of the study included a comparatively small sample size with lesser patients in the low disease activity group. ROS production was measured by the NBT test which is currently surpassed by newer methods like Dihydrorhodamine (DHR) test by flow cytometry or other immunofluorescence-based assays. NBT test used in this study denotes NOX induced cytoplasmic ROS production, but mitochondrial ROS also has tremendous role in lupus pathogenesis as evident by recent literature. It was a cross-sectional study; prospective study design with serial measurements of ROS would make it more reliable and informative. Upcoming studies based on single cell transcriptomics will find out elaborate details of cytoplasmic and mitochondrial ROS production in lupus patients.^18^

## CONCLUSION

The present study showed that on stimulation, lupus patients produce more ROS than healthy controls. Besides, basal ROS generation was also significantly higher in patients with high disease activity than those with low disease activity. Further studies are needed to explore the mechanisms underlying the increased neutrophil oxidative metabolism and its immunologic consequences in the pathophysiology of systemic lupus erythematosus.
